# Outcomes in Hepatitis C Positive Liver Transplantation: Timing of Direct-Acting Antiviral Treatment and Impact on Graft Fibrosis

**DOI:** 10.3390/v13091831

**Published:** 2021-09-14

**Authors:** Jennifer Wellington, Andrew Ma, Shyam Kottilil, Bharath Ravichandran, Jennifer Husson, David Bruno, Eleanor Wilson

**Affiliations:** 1 Division of Gastroenterology and Hepatology, University of Maryland School of Medicine, Baltimore, MD 21201, USA; 2 Department of Internal Medicine, University of Maryland School of Medicine, Baltimore, MD 21201, USA; ama@som.umaryland.edu; 3 Division of Clinical Care and Research, Institute of Human Virology, University of Maryland School of Medicine, Baltimore, MD 21201, USA; skottilil@ihv.umaryland.edu (S.K.); jhusson@ihv.umaryland.edu (J.H.); eleanor.wilson@ihv.umaryland.edu (E.W.); 4 Department of Pharmacy, University of Maryland School of Medicine, Baltimore, MD 21201, USA; bravichandran@umm.edu; 5 Division of Transplant Surgery, University of Maryland Medical Center, Baltimore, MD 21201, USA; dbruno@som.umaryland.edu

**Keywords:** liver transplant, hepatitis C Virus, graft fibrosis

## Abstract

Liver transplantation for hepatitis C virus (HCV)-related disease has the lowest five-year graft survival among all liver transplant recipients. Graft failure due to accelerated fibrosis from unrestrained HCV replication is common. Optimal timing of HCV treatment with direct-acting antiviral agents remains unknown. We compared HCV liver transplant recipients successfully treated for HCV before transplant to those treated within 1 year of transplant to determine if graft fibrosis, measured by Fib-4 scores, differs with timing of treatment. Fib-4 scores less than or equal to 1.45 defined minimal fibrosis and greater than 1.45 defined greater than minimal fibrosis. We identified 117 liver transplant recipients: 52 treated before transplantation and 65 treated within 1 year of transplantation. Overall, 34% of recipients had minimal fibrosis, and the likelihood of having minimal fibrosis following treatment and liver transplantation did not differ by timing of treatment. The odds ratio of having greater than minimal fibrosis was 0.65 (95% CI 0.30, 1.42) among those treated within 1 year after transplantation compared to those treated before transplantation (*p*-value 0.28). Importantly, nearly 2/3 of these patients had evidence of fibrosis progression one year after sustained virologic response, supporting recommendations for early antiviral-based treatment to prevent accumulation of HCV-related disease.

## 1. Introduction

Liver transplant (LT) graft survival for those transplanted for HCV-related liver disease is the lowest amongst all LT recipients [1]. Many studies have attributed poor graft survival to the immediate and aggressive re-infection of the graft and accelerated fibrosis that occur within the first few years after surgery [2,3,4]. Recently, studies have shown improved patient survival for those who are HCV RNA negative at time of transplant [5]. The advent of highly effective direct acting antiviral (DAA) treatment offers decompensated cirrhotic patients, previously known as difficult-to-treat in the interferon-era, a chance at treatment with response rates approaching 90% [6,7,8,9]. Sustained viral response (SVR) is difficult to achieve in decompensated disease and in this setting, viral suppression for thirty days before transplant may be enough to prevent graft re-infection and potentially lead to better graft outcomes [9].

Controversy persists regarding treatment of these patients as many ultimately go on to transplant, and the impact of treatment on graft fibrosis has not yet been studied in this group. As a result, current practice guidelines from the Infectious Disease Society of America (IDSA), American Association for the Study of Liver Disease (AASLD) and European Association for the Study of Liver Disease (EASL) recommend against treatment for HCV in severe decompensated disease, specifically those with MELD scores greater than 18–20, as they are better served by transplantation and treatment after transplantation may be more effective [10,11,12]. The effect that treatment may have on graft fibrosis for those who were treated before transplant compared to those treated after liver transplant remains unknown. Previous studies have identified several independent risk factors associated with increased fibrosis. Recipient characteristics including age at transplant, sex, race, diabetes, coinfection and donor characteristics including age, HCV exposure, cold ischemic time and donation after cardiac death have consistently been reported in the literature to influence fibrosis progression [3,13,14]. The relationship between these factors and graft fibrosis in the setting of HCV treatment has not been previously reported. Outcomes of recent studies have mainly emphasized rates of SVR from DAA therapy, not graft fibrosis, and length of follow up is limited given the novelty of these drugs. 

Our study aims to assess the impact of DAA treatment on graft fibrosis in patients treated before or after LT by utilizing the Fib-4 score. The Fib-4 score, a non-invasive indirect measure of fibrosis, has been previously validated for use in this population [15,16,17]. A recent study found that the Fib-4 score accurately estimated fibrosis regression among decompensated cirrhotic patients after antiviral therapy [16]. We hypothesized that treatment with DAAs after transplant may prevent graft fibrosis, resulting in lower Fib-4 scores compared to those treated prior to transplant. 

## 2. Materials and Methods

### 2.1. Patient Cohort

**Figure 1:**
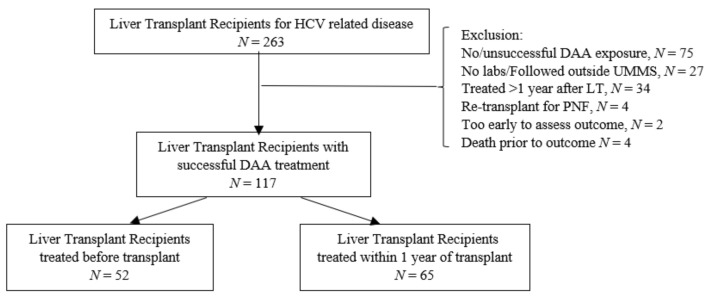
Liver transplant recipient study selection. From the 263 potentially eligible patients, 117 met the inclusion criteria. Patients were divided into two groups for analysis: liver transplant recipients treated before transplant, *N* = 52 and liver transplant recipients treated within one year of transplant, *N* = 65. HCV, hepatitis C virus; DAA, direct acting antiviral therapy; UMMS, University of Maryland Medical System; PNF, primary non-function.

We identified 263 HCV positive patients greater than 18 years of age who received a primary, secondary or simultaneous liver-kidney transplant from a living or deceased donor for HCV-related end stage liver disease (ELSD) or hepatocellular carcinoma (HCC) at the University of Maryland Medical System between 2012–2017 in a single-center retrospective cohort analysis. Liver transplant recipients were eligible for our study if they achieved SVR following DAA treatment. Transplant recipients with any of the following were excluded: primary graft non-function, (*N* = 4); no available laboratory values/followed outside of the University of Maryland Medical System after transplant, (*N* = 26); no/unsuccessful DAA exposure, (*N* = 75), too early to assess outcome, *(N* = 2); death prior to outcome, (*N* = 4) and DAA treatment after one year following LT, (*N* = 34). Recipients of HCV positive donor organs and those with treated co-infections including HIV and HBV were not excluded. Transplant recipients meeting study criteria were stratified into two groups based on timing of antiviral treatment before LT (*N* = 52) and within one year after LT (*N* = 65). Detailed information about the selection of the study cohort is presented in Figure 1. The Institutional Review Board at the University of Maryland Medical System determined that our study was exempt from review.

### 2.2. Antiviral Treatment

The DAA regimen, indication for treatment, dose and duration of therapy were determined by the transplant hepatologist as standard of care independent of this study. All patients received a combination of non-interferon based DAAs available during the study time frame, with or without the addition of ribavirin. Successful treatment was determined by achievement of SVR, defined as undetectable HCV RNA by COBAS^®^ TAQMAN^®^ HCV TEST v2.0, Roche Diagnostics, Indianapolis, Indiana, USA at 12 weeks after end of treatment (SVR_12_) and one year after SVR_12_ in all patients.

### 2.3. Main Study Outcomes 

The Fib-4 score was used in this study as a surrogate measure for liver graft fibrosis. A Fib-4 score, calculated by (age × AST)/(platelet × √ALT), of less than or equal to 1.45 correlates to the favorable outcome of minimal fibrosis with a NPV of 90% and sensitivity of 85% and a score greater than or equal to 3.25 corresponds to advanced fibrosis with a PPV of 67% and a specificity of 94% [15,17]. Fib-4 scores were calculated at 1 year after transplant if treated before transplantation and 1 year after sustained virologic response if treated after transplantation. The Fib-4 score modeled in this study is a score greater than 1.45, corresponding to greater than minimal fibrosis. 

### 2.4. Database 

A database (Microsoft Excel 2016, Microsoft Corporation, Redmond, WA, USA) was created containing liver transplant recipient and donor characteristics from data available by University of Maryland Medical Center EMR and UNOS transplant candidate registration.

### 2.5. Potential Confounders

Potential confounders included transplant recipient and donor factors previously identified in the literature. Specific transplant recipient factors considered include recipient’s age at transplant, sex, race, year of transplant, graft rejection, and the diagnosis of diabetes mellitus, HCC, HIV, HBV, or CMV. Specific donor factors considered include donor’s age, HCV diagnosis, graft cold ischemic time and cause of death.

### 2.6. Immunosuppression

At our institution, all liver transplant recipients that are hepatitis C positive receive a standard immunosuppression protocol consisting of a three-day steroid taper, mycophenolate 1000 mg total daily dose or equivalent, and tacrolimus with a goal of 6–8 ng/mL. Simultaneous liver kidney transplant recipients follow this same protocol, with the exception of receiving mycophenolate 2000 mg total daily dose.

### 2.7. Data Analysis

Statistical analyses were performed using SAS software (version 9.4; SAS Institute Inc., Cary, NC). Baseline characteristics are described as frequencies and percentages or medians with IQR for each group in Table 1. Categorical variables were compared using *χ*^2^-test or Fischer’s exact test when indicated. Continuous variables and variables not distributed normally were compared using the Wilcoxon rank sum test. Simple imputation methods were used for those who had laboratory values available within 60 days of the study endpoint time frame to calculate Fib-4 scores. The association of the treatment group and the Fib-4 score was analyzed by logistic regression; *p*-values < 0.05 were considered statistically significant.

## 3. Results

### 3.1. Patient Characteristics at Time of Transplantation

Overall, 117 transplant recipients met eligibility criteria and were stratified into two groups based on timing of DAA treatment: 52 recipients treated before LT and 65 recipients treated within 1 year following LT. Clinical characteristics are shown in Table 1. The majority of the total study sample were white, 58%, and male, 72%, which was similar between treatment groups. The overall median age at transplant was 60 years (56–64) which varied significantly among treatment groups, *p* = 0.04. The most common HCV genotype was 1a. Those treated prior to LT were more likely to be diabetic or have HCC. Acute cellular rejection was defined by the presence of rejection on liver biopsy which was present in 29% of those treated before LT and 43% of those treated within one year of LT with no difference between groups, *p* = 0.11. No recipient had evidence of chronic rejection within the follow up period.

### 3.2. Donor Characteristics

The donor characteristics are shown in Table 1 as obtained from the UNOS donor registry. Overall, the donors had a median age of 44 years (30–56). HCV antibody positive donor grafts were transplanted in 40% of recipients without any difference in distribution between groups, *p* = 0.06 as shown in Table 2. Of note, donor HCV viremia status by nucleic acid testing (NAT) was not universally available during the study period.

### 3.3. Confounding

Several variables shown in Table 1 were significantly different among treatment groups, but adjusting individually for these variables with *p*-value < 0.2 did not substantially adjust the crude odds ratio and therefore confounding was not identified. There was no effect measure modification between male and female LT recipients.

### 3.4. Graft Fibrosis

Overall, 34% of recipients had a Fib-4 score ≤ 1.45, indicating minimal fibrosis as shown in Table 3. The odds ratio of having greater than minimal fibrosis (Fib-4 score > 1.45) was 0.65 (95% CI 0.30, 1.42) among those treated within 1 year after transplantation compared to those treated before transplantation, *p* = 0.28.

## 4. Discussion

In this cohort study, we compared two distinct groups of HCV positive LT recipients to determine if graft fibrosis differs with the timing of successful HCV treatment and LT. We found that LT recipients that were treated within the first year after LT had 33% reduced odds of having greater than minimal graft fibrosis compared to those treated before LT; however, this result did not achieve statistical significance. This finding is compelling in that it shows no difference in fibrosis between those treated before transplant or one year after. Enhanced tolerability, success of SVR, and wide availability of DAA therapy for these patients have likely contributed to the reduced fibrosis seen in these patients. Clinically, these findings support the recommendations for early DAA treatment in the peri-transplant setting. 

Our study endpoint of fibrosis after DAA treatment in the peri-transplant setting has not been previously studied given the novelty of these drugs, low numbers of subjects and limited follow up duration. HCV eradication has been shown to lead to reduced progression and even reversal of liver fibrosis however these outcomes were not generalizable to the liver transplant population as they were often excluded from these studies [18]. The outcomes of many landmark studies regarding DAA-based therapy outcomes have been centered around achievement of SVR, drug tolerability and safety in transplant patients and survival of liver grafts and recipients [6,19,20,21,22,23]. As DAA-based treatments have become more mainstream therapy and access to treatment has improved due to decreased costs and broadened insurance coverage, a wealth of data has emerged. We studied two distinct groups of HCV positive LT recipients to determine if graft fibrosis differs with the timing of successful HCV treatment and LT. 

A compelling study by Neumann et al. concluded that LT recipients with recurrent HCV infection had rapidly progressive graft fibrosis within the first three years after LT [13]. This study was the first of its kind to demonstrate that a histopathological fibrosis stage greater than 2 within the first year of LT led to a 15-fold increased risk of HCV-related graft loss [13]. Notably, three patients (4.4%) in this study had no fibrosis within the first year of LT and had higher graft survival rates than those with advanced fibrosis [13]. In our study, 35% (40/117) of our LT recipients had minimal fibrosis overall. Thus, it is likely that treatment before or within one year of LT reduces the development of fibrosis by eradicating HCV, which may improve graft survival. 

Donor characteristics play an important role in the long-term outcomes for LT recipients. Younger donor age has been consistently associated with improved liver graft outcomes, including fibrosis [13,24,25]. Our transplant center’s average donor age has been decreasing in recent years, similar to the nationwide trend, due in part to increased deaths from anoxia attributed to the opioid epidemic [1,26,27]. Organs from these high-risk individuals have saturated the donor pool with HCV positive organs which are frequently discarded [28]. Before a suitable cure was available, HCV positive donor tissue had been shown to lead to increased fibrosis lending to poor outcomes in LT recipients [13]. Importantly however, 40% of our study population received HCV antibody positive grafts from deceased donors, and this was not associated with increased fibrosis (*p* = 0.44). This provides initial evidence to support recent interest in the transplantation of HCV positive donor tissue in HCV negative recipients, which needs further study and evaluation of long term outcomes [27,29].

Variation in the timing of DAA treatment among these patients was determined by several factors that cannot be completely accounted for in this observational study. To overcome this limitation, baseline Fib-4 scores were compared at baseline and were not statistically significant between treatment groups. Variability in timing of DAA treatment has been a significant limitation in the literature and underscores the importance of updated guidelines to encourage early treatment as a standard of care. 

In conclusion, our cohort of LT recipients with HCV provides evidence that successful treatment of HCV either before or within one year of LT results in no difference in the likelihood of minimal fibrosis after treatment. More than one-third of these patients had minimal evidence of graft fibrosis one year after SVR, which supports current guidelines that recommend early DAA-based treatment to prevent accumulation of HCV-related disease.

## Figures and Tables

**Table 1:** Liver transplant recipient and donor clinical characteristics by treatment group, overall *N* = 117.

Variable:	Overall *N* = 117	Treated Before *N* = 52	Treated < 1 Year *N* = 65	*p*-Value
Age at Transplant, Median (IQR)	60 (56–64)	61 (57–64.5)	58 (54–62)	0.04
Sex (Col %)				0.89
Male, *N* (%)	84 (71.8)	37 (71.2)	47 (72.3)	
Female, *N* (%)	33 (28.2)	15 (28.9)	18 (27.7)	
Race				0.19
White, *N* (%)	68 (58.1)	32 (61.5)	36 (55.4)	
Black, *N* (%)	47 (40.2)	18 (34.6)	29 (44.6)	
Other, *N* (%)	2 (1.7)	2 (3.9)	0	
Diabetes, *N* (%)	24 (20.7)	14 (26.9)	10 (15.6)	0.33
HCC, *N* (%)	65 (55.6)	32 (61.5)	33 (50.8)	0.24
Co-infection				
HBV core Ab *N* (%)	49 (41.9)	20 (38.5)	29 (44.6)	0.50
CMV Ab *N* (%)	78 (66.7)	33 (63.5)	45 (69.2)	0.51
HIV *N* (%)	3 (2.6)	0	3 (4.6)	0.25
HCV Genotype				0.79
1a *N* (%)	82 (75.9)	31 (72.1)	51 (78.5)	
1b *N* (%)	16 (14.8)	7 (16.3)	9 (13.9)	
2 *N* (%)	5 (4.6)	3 (7.0)	2 (3.1)	
3 *N* (%)	5 (4.6)	2 (4.7)	3 (4.6)	
MELD score, Avg (SD)	20 (10)	17 (8)	22 (11)	<0.01
Baseline Fib-4, Median (IQR)	1.84 (1.31–2.83)	1.75 (1.30–2.76)	1.86 (1.32–3.18)	0.47
Transplant year				<0.01
2013 *N* (%)	4 (3.4)	0	4 (6.2)	
2014 *N* (%)	18 (15.4)	1 (1.9)	17 (26.2)	
2015 *N* (%)	44 (37.6)	20 (38.5)	24 (36.9)	
2016 *N* (%)	38 (32.5)	20 (38.5)	18 (27.7)	
2017 *N* (%)	13 (11.1)	11 (21.2)	2 (3.1)	
Rejection	43 (36.7)	15 (28.8)	28 (43.1)	0.11
Donor Characteristics				
Age of Donor, Median (IQR)	44 (30–56)	46.5 (32–55)	37 (30–56)	0.48
Living *N* (%)	9 (7.7)	3 (5.8)	6 (9.2)	0.49
HCV Ab *N* (%)	47 (40.2)	16 (30.8)	31 (47.7)	0.06
CIT, hours avg (SD)	4.1 (1.4)	4.1 (1.2)	4.1 (1.5)	0.88
DCD *N* (%)	15 (12.8)	9 (17.3)	6 (9.2)	0.19

Percentages shown are column percentages. HCC, hepatocellular carcinoma; HBV Core Ab, hepatitis B core antibody positive; CMV Ab, cytomegalovirus antibody positive, HIV, human immunodeficiency virus antibody positive; MELD, Model for End Stage Liver Disease score; HCV ab, hepatitis C antibody positive; CIT, cold ischemic time in fraction of hours; DCD, donation after cardiac death.

**Table 2:** Association between liver transplant recipient and donor characteristics and Fib-4 score <1.45 corresponding to little or minimal fibrosis or >1.45 indicating at least moderate fibrosis or greater.

Variable:	FIB-4 ≤ 1.45 *N* = 40	FIB-4 > 1.45 *N* = 77	*p*-Value
Age at Transplant, Median (IQR)	60 (54.5–63.5)	60 (56–64)	0.41
Sex (Row %)			0.46
Male, *N* (%)	27 (32.1)	57 (67.9)	
Female, *N* (%)	13 (39.4)	20 (60.6)	
Race			0.32
White, *N* (%)	20 (29.4)	48 (70.6)	
Black, *N* (%)	28 (59.6)	19 (40.4)	
Other, *N* (%)	1 (50.0)	1 (50.0)	
Diabetes, *N* (%)	8 (33.3)	16 (66.7)	0.89
HCC, *N* (%)	21 (32.3)	44 (67.7)	0.69
Co-infection			
HBV core Ab *N* (%)	16 (32.7)	33 (67.3)	0.77
CMV Ab *N* (%)	26 (33.3)	52 (66.7)	0.78
HIV *N* (%)	1 (33.3)	2 (66.7)	0.97
HCV Genotype			0.27
1a *N* (%)	32 (39.1)	50 (60.9)	
1b *N* (%)	5 (31.2)	11 (68.8)	
2 *N* (%)	0	5 (100)	
3 *N* (%)	1 (20.0)	4 (80.0)	
MELD score, Avg (SD)	21 (11)	19 (9)	0.30
Baseline Fib = 4 score, Median (IQR)	1.32 (1.04–1.71)	2.17 (1.66–3.65)	0.98
Transplant year			0.55
2013 *N* (%)	0	4 (100)	
2014 *N* (%)	8 (44.4)	10 (55.6)	
2015 *N* (%)	17 (38.6)	27 (61.4)	
2016 *N* (%)	12 (31.6)	26 (68.4)	
2017 *N* (%)	3 (23.1)	10 (76.9)	
Rejection	13 (32.5)	30 (38.9)	0.49
Donor Characteristics			
Age of Donor, Median (IQR)	36 (28.5–50.5)	49 (31–58)	0.03
Living *N* (%)	1 (11.1)	8 (88.9)	0.16
HCV Ab *N* (%)	18 (38.3)	29 (61.7)	0.44
CIT, hours avg (SD)	4.3 (1.2)	4.0 (1.42)	0.27
DCD *N* (%)	4 (26.7)	11 (73.3)	0.51

Percentages shown are row percentages. HCC, hepatocellular carcinoma; HBV Core Ab, hepatitis B core antibody positive; CMV Ab, cytomegalovirus antibody positive, HIV, human immunodeficiency virus antibody positive; MELD, Model for End Stage Liver Disease score; HCV ab, hepatitis C antibody positive; CIT, cold ischemic time in fraction of hours; DCD, donation after cardiac death.

**Table 3:** Odds ratio and 95% interval of the association between treatment group and Fib-4 score <1.45 corresponding to little or minimal fibrosis or >1.45 indicating at least moderate fibrosis or greater. Odds ratio for Fib-4 score >1.45 reported.

	FIB-4 ≤ 1.45 *N* (%)	FIB-4 > 1.45 *N* (%)	OR (95% CI)	*p*-Value
Treated Before	15 (28.8)	37 (71.2)	Ref	
Treated < 1 year after	25 (38.5)	40 (61.5)	0.65 (0.30–1.42)	0.28

Percentages shown are row percentages.

## Data Availability

Data available upon request.

## Abbreviations

AASLD	American Association for the Study of Liver Disease
ALT	Alanine aminotransferase
AST	Aspartate aminotransferase
CIT	Cold ischemic time
CMV	Cytomegalovirus
DAA	Direct acting antivirals
DCD	Donation after cardiac death
EASL	European Association for the Study of Liver Disease
Fib-4	Fibrosis-4 index
HBV	Hepatitis B virus
HCC	Hepatocellular Carcinoma
HCV	Hepatitis C virus
HIV	Human immunodeficiency virus
IDSA	Infectious Disease Society of America
LT	Liver transplant
MELD	Model for End Stage Liver Disease
NAT	Nucleic Acid Test
PNF	Primary non-function
RNA	Ribonucleic Acid
SVR	Sustained viral response
UMMS	University of Maryland Medical System
UNOS	United Network for Organ Sharing
